# The Formation of Stromules *In Vitro *from Chloroplasts Isolated from *Nicotiana benthamiana*

**DOI:** 10.1371/journal.pone.0146489

**Published:** 2016-02-03

**Authors:** Jonathan Ho, Steven M. Theg

**Affiliations:** Department of Plant Biology, University of California Davis, Davis, California, United States of America; Arizona State University, UNITED STATES

## Abstract

Stromules are stroma-containing tubules that have been observed to emanate from the main plastidic body *in vivo*. These structures have been shown to require cytoskeletal components for movement. Though numerous studies have shown a close association with the endoplasmic reticulum, nucleus, mitochondria, and other plastids, the mechanism of formation and their overall function remain unknown. A limiting factor in studying these structures has been the lack of a reconstituted system for *in vitro* stromule formation. In this study, stromule formation was induced *in vitro* by adding a plant extract fraction that is greater than 100 kDa to a population of isolated chloroplasts. Kinetic measurements show that stromule formation occurs within ~10 seconds after the addition of the plant extract fraction. Heat inactivation and apyrase treatment reveal that the stromule stimulating compound found in the extract fraction is a protein or protein complex 100 kDa or greater. The formation of the stromules *in vitro* with isolated chloroplasts and a concentrated fraction of cell extract opens an avenue for the biochemical dissection of this process that has heretofore been studied only *in vivo*.

## Introduction

Plastids are double membrane enclosed organelles that carry out essential functions in plants. These tasks range from manufacturing and storing starch in leucoplasts to conversion of light energy into sugars as seen in chloroplasts. The adoption of fluorescent proteins by cell biologists has allowed researchers to monitor the structural changes that organelles undergo *in vivo*. While tubular projections emanating from plastids had been noted as long as 107 years ago [1], they did not receive much attention until they were observed using a stromal protein fused to GFP [2]. The term "stromule" was coined to describe these stroma-containing tube-like projections [3]. Stromules were observed to be 0.35 μm to 1 μm in diameter and up to ~200 μm in length [2, 4–6]. Further studies showed that actin microfilaments and myosin IX appear to be required for the movement of these structures *in vivo* [6–9]. Plastids without chlorophyll and those found in areas of low plastid density display a higher frequency of stromule formation [3, 10]. Plants subjected to water, temperature, or pathogenic stresses also exhibit a higher frequency of stromules [11–16], pointing to their possible role in stress signaling. Close contact with mitochondria, the endoplasmic reticulum, nuclei, and other plastids further suggests that stromules might function as conduits for the exchange of metabolites or genetic information [6, 8, 14–18], although this point remains somewhat controversial [19]. Despite many years of research activity, the mechanism of stromule formation remains unknown.

To date, most of the studies investigating stromules have been performed *in vivo* or on epidermal peels. In this study, we report the establishment of a robust assay for *in vitro* stromule formation using isolated chloroplasts. Herein we provide results showing a dependency of stromule formation on a protein or protein complex larger than 100 kDa located in an isolated plant extract fraction.

## Materials and Methods

### Plant growth conditions

*Nicotiana benthamiana* were grown in growth chambers with conditions set at 20°C with 16 hrs light cycle of 100 μmol photons/m^2^/sec at 60% humidity. Plants were germinated and planted following the protocol in [20].

### Chloroplast isolation

*N*. *benthamiana* plants that had been transformed with NRIP1 fused to cerulean (gift from S.P. Dinesh-Kumar) were used for all experiments [12]. Plants were grown for 9–12 weeks and harvested for chloroplasts. The plastid isolation protocol is similar to the chloroplast isolation procedure for *Pisum sativum* [21]. Briefly, leaves of *N*. *benthamiana* were blended with a grinding buffer (50 mM Tricine- KOH, pH 8.0, 330 mM sorbitol, 1 mM MnCl_2_, 1 mM MgCl_2_, 2 mM Na_2_EDTA, and 0.1% BSA). The slurry was passed through 2 layers of cheesecloth and centrifuged at 3,000 x *g* for 5 minutes. The soluble fraction was saved as the plant extract fraction, while the pellet was resuspended and placed onto a continuous Percoll gradient and centrifuged for 10 minutes. The intact chloroplasts were removed from the Percoll gradient and washed twice with a chloroplast storage buffer (330 mM Sorbitol, 50 mM Hepes-KOH, pH 8) and stored on ice in the dark until used.

### Plant extract preparation

The plant extract fraction collected after the initial centrifugation step was centrifuged again at 3,300x g for 5 minutes to remove any additional thylakoid particles. The cell extract was then concentrated 40-fold using 100 kDa mwco Amicon centrifugal filtration devices (EMD Millipore, Billerica, MA), quickly frozen in liquid nitrogen, and stored in a -80°C freezer. The 100 kDa≥ x≥10 kDa fraction was created by taking the flowthrough from the greater than 100 kDa concentration step and passing that through a 10 kDa mwco Amicon centrifugal filter device (EMD Millipore, Billerica, MA). The filtrate was also concentrated 50 fold. Heat- inactivated extract was made by placing the greater than 100 kDa plant extract fraction into a 70°C water bath for 10 minutes. The aggregates were removed by a 2 minute centrifugation at 16,000 x *g* in a table top centrifuge. The extract was then stored on ice for two hours before use. Fractionation of the whole-cell extract was carried out by centrifuging the concentrated extract at 16,000 x *g* for 10 minutes at 4°C. The supernatant was then centrifuged at 100,000 x *g* for 1 hour at 4°C using a TL-100 Ultracentrifuge (Beckman Coulter Inc., Brea, CA). The supernatant was collected and stored on ice before use. The pellet was resuspended in chloroplast storage buffer and stored on ice until further use. Apyrase-treated extract was prepared by incubating apyrase (Sigma-Aldrich, St. Louis, MO) with the extract at a final concentration of 125 units/ml in 200 μL for 2 hours on ice.

### Microscopy and quantitation

A homemade perfusion chamber consisting of a chamber milled from a 1.8 mm x 100 mm x 1.1 mm piece of polycarbonate and a 50 x 22 mm coverslip (Fisher Scientific Houston, TX) were used for all of the imaging studies. The working perfusion chamber was assembled by mounting a poly-L-lysine (Sigma-Aldrich St. Louis, MO) coated cover slip onto the bottom of the chamber using silicone grease (Beckman Coulter Inc., Brea, CA). Isolated chloroplasts (0.5 μg Chl) were placed into each chamber and centrifuged for 15 minutes at 60 x *g* using a GS-6KR swinging bucket centrifuge (Beckman Coulter Inc., Brea, CA) at 10°C. The samples were then observed under the microscope. Images were taken using a Zeiss LSM 710 confocal microscope (Zeiss, Oberkochen, Germany). The Cerulean and chlorophyll were excited using the 458 nm laser. 200 μL of treatment solutions, depending on the reaction, was added to each chamber. Image compilation and analysis were performed using Fiji software. Images captured from the cerulean channel in the microscope were artificially colored with a grey LUT to enhance contrast.

## Results

### Plant extract stimulates stromule formation in isolated chloroplasts

**Fig 1:**
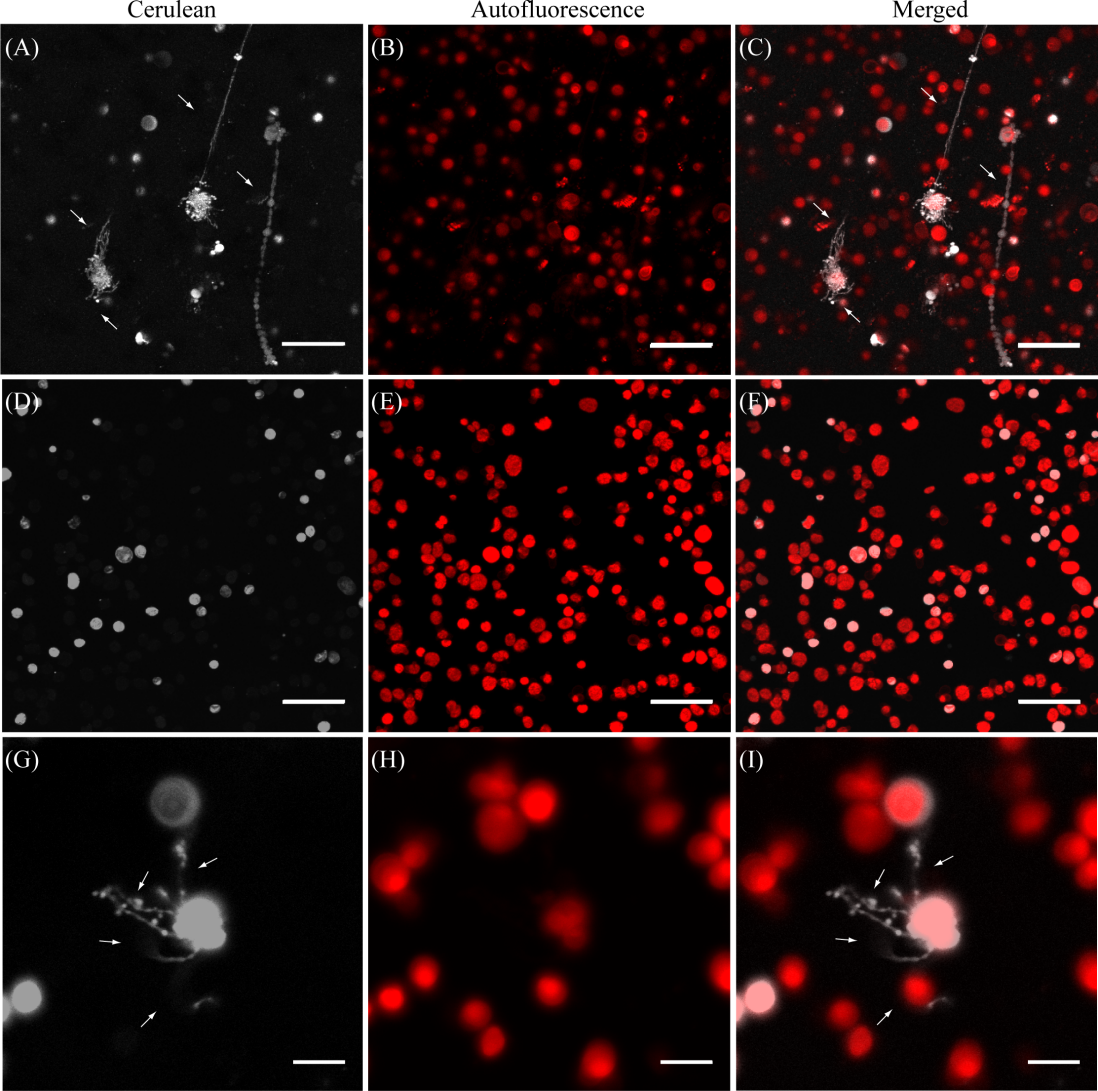
*In vitro* stromule formation requires plant extract. A-C: A concentrated cell extract fraction was added to fixed chloroplasts and observed under a microscope. D-F: Isolated chloroplasts treated with a solution of 0.5% BSA, 2 mM ATP and 200 μM GTP. G-I: A zoomed in image of a stimulated chloroplast shows multiple stromules with nodules emanating from a single plastid. Scale bars in panels A–D correspond to 30 μm; those in G–I, 10 μm. All images were organized by showing the cerulean (grey), autofluorescence (red), and merged images from left to right. White arrows indicate stromules. The total elapsed time of the recording was 10 minutes and 23 seconds.

*N*. *benthamiana* plants transformed with a Cyan Fluorescent Protein (Cerulean) fused to the C- terminus of NRIP1, a chloroplast stromal protein, were placed behind its native promoter and used as a source for the isolation of intact chloroplasts [12]; the Cerulean-tagged protein serves as a stromal marker. Isolated chloroplasts were centrifuged onto a poly-L-lysine-coated coverslip in a homemade perfusion chamber, incubated for 30 minutes with various treatments, and monitored for morphological changes under a confocal microscope. Chloroplasts that were treated with a concentrated plant extract fraction prepared by using a 100 kDa molecular weight cutoff (mwco) filter displayed a significant change in their morphology (Fig 1A–1C). The images from the Cerulean channel captured several chloroplasts with protrusions containing stroma emanating from the plastid (Fig 1A). The chlorophyll autofluorescence channel showed that the thylakoids remained spherical and at the main body of the chloroplasts (Fig 1B). Chlorophyll autofluorescence was not observed in any of the protrusions, indicating that the structures contained only the plastid envelope membranes and stroma. The merged image shows that the extensions contained only stroma and that these structures were stromules (Fig 1C). The chloroplasts exhibited a variety of morphologies following the addition of the plant extract. We observed several chloroplasts that had multiple stromules forming off of a single body with lengths that varied up to ~ 40 μm. The longest projections seen in our studies was 109 *μm* (S1 Movie). It has been reported that stromules can reach up to 220 μm in length [10]. Other chloroplasts in our experiments showed only one large stromule, while a few did not display any stromules in our images. Higher resolution images of a stimulated chloroplast showed nodules that formed along the side or at the end of the stromules (Fig 1G–1I). The diameter of these structures were typically 1 μm with occasional nodules widening up to 2 μm. Other studies performed in *Lucopersicon esculentum* chromoplasts and chloroplasts [5, 6] have also reported "bead-like" sacks along the stromule which were thought to arise from the redistribution of stromal contents as a result of excessive stretching. The *in vitro*-formed stromules in our experiments also displayed occasional branching (S2 Movie). The plant extract stimulated stromule formation in 40.1% ± 19.9% of the intact chloroplasts (n = 15). The smallest stromule included in our quantitation was 0.8 μm in length.

To test whether a substance in the plant extract fraction stimulated stromule formation in our isolated chloroplasts, plastids were incubated with the storage buffer supplemented with 0.5% BSA, 2 mM ATP and 200 μM GTP (Fig 1D–1F). The isolated chloroplasts maintained a smooth spherical shape (Fig 1D) without any long tube-like projections emanating from the main body. A few chloroplasts exhibited very short and transient protrusions mostly less than 0.5 microns in length. The addition of BSA, ATP, and GTP only showed a 2.0% ± 2.3% stimulation (n = 3). These results indicate that the plant extract is responsible for the induction of stromule formation.

**Fig 2:**
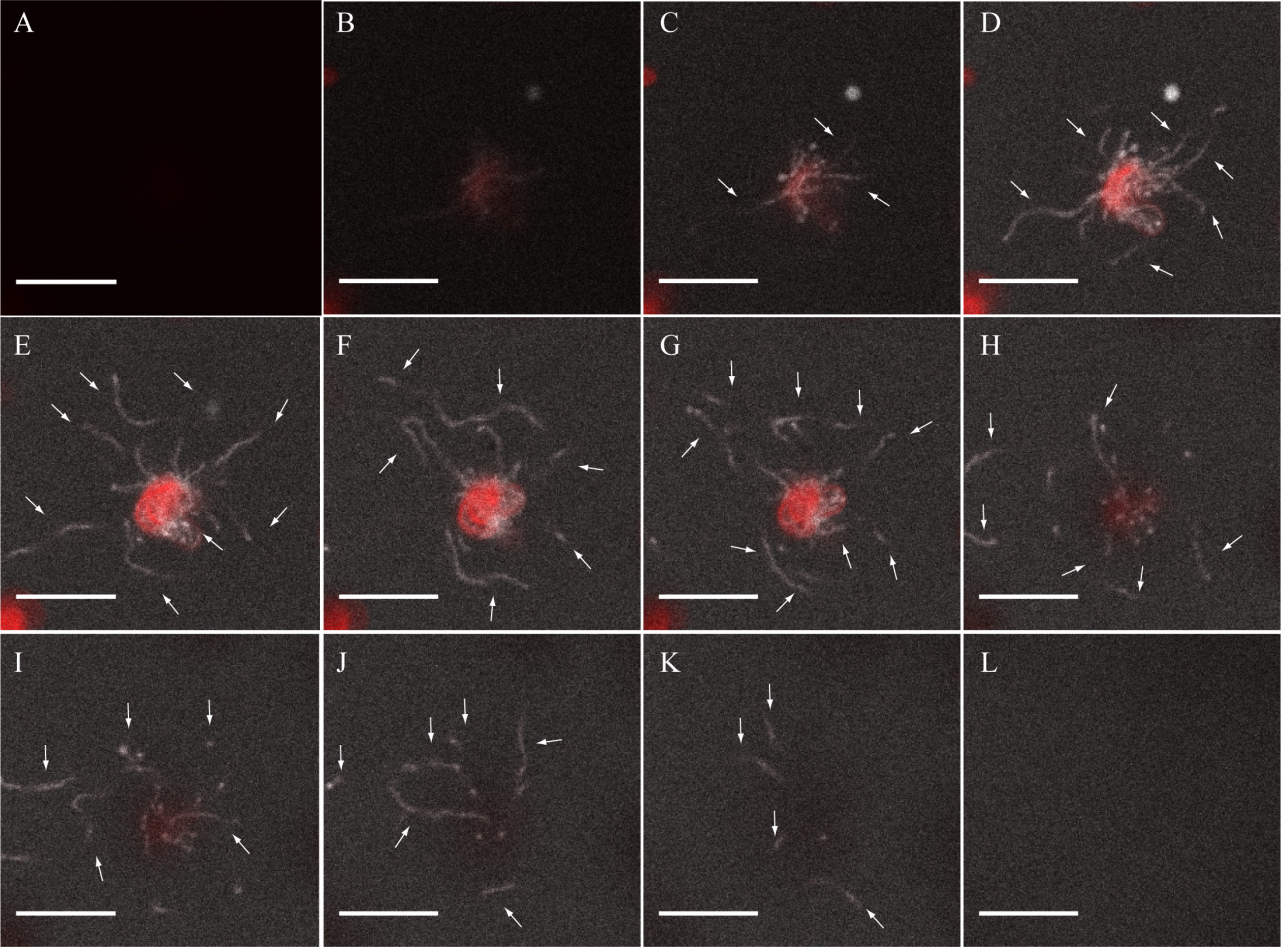
Stromules are dynamic structures that also move throughout the z planeA-L: An image sequence through the z axis of two chloroplasts that have been stimulated to form stromules. The scale bar corresponds to 10 μm; the 12 panels cover 19.8 μm in the z direction. The white arrows mark stromule branches.

The stromules that formed in our *in vitro* experiments were not static distortions of the envelope membranes. This is seen in time-lapsed videos that capture the dynamic movement of these structures over the course of 10 minutes (S3 Movie). The stromules in S3 Movie initially appear to contact other chloroplasts and move around a thylakoid released from a broken plastid. Over the course of 10 minutes, the stromules can be seen to retract back to the main body of the chloroplast, and at the end appear to have been reabsorbed. Palpitations can be seen as the *in vitro-*stimulated stromules float in the reaction buffer, suggesting that they are under little or no tension. Not only are the stromules dynamic in the sense that they are capable of moving in two dimensions, the stimulated chloroplasts display multiple protrusions at different planes along the z—axis (Fig 2A–2L), reaching ~20 μm in the z direction as well. The dynamic structures that formed as a result of the incubation of isolated chloroplasts with plant extract prompted us to investigate the initial stages of stromule formation.

### Stromule formation occurs rapidly after the addition of cell extract

**Fig 3:**
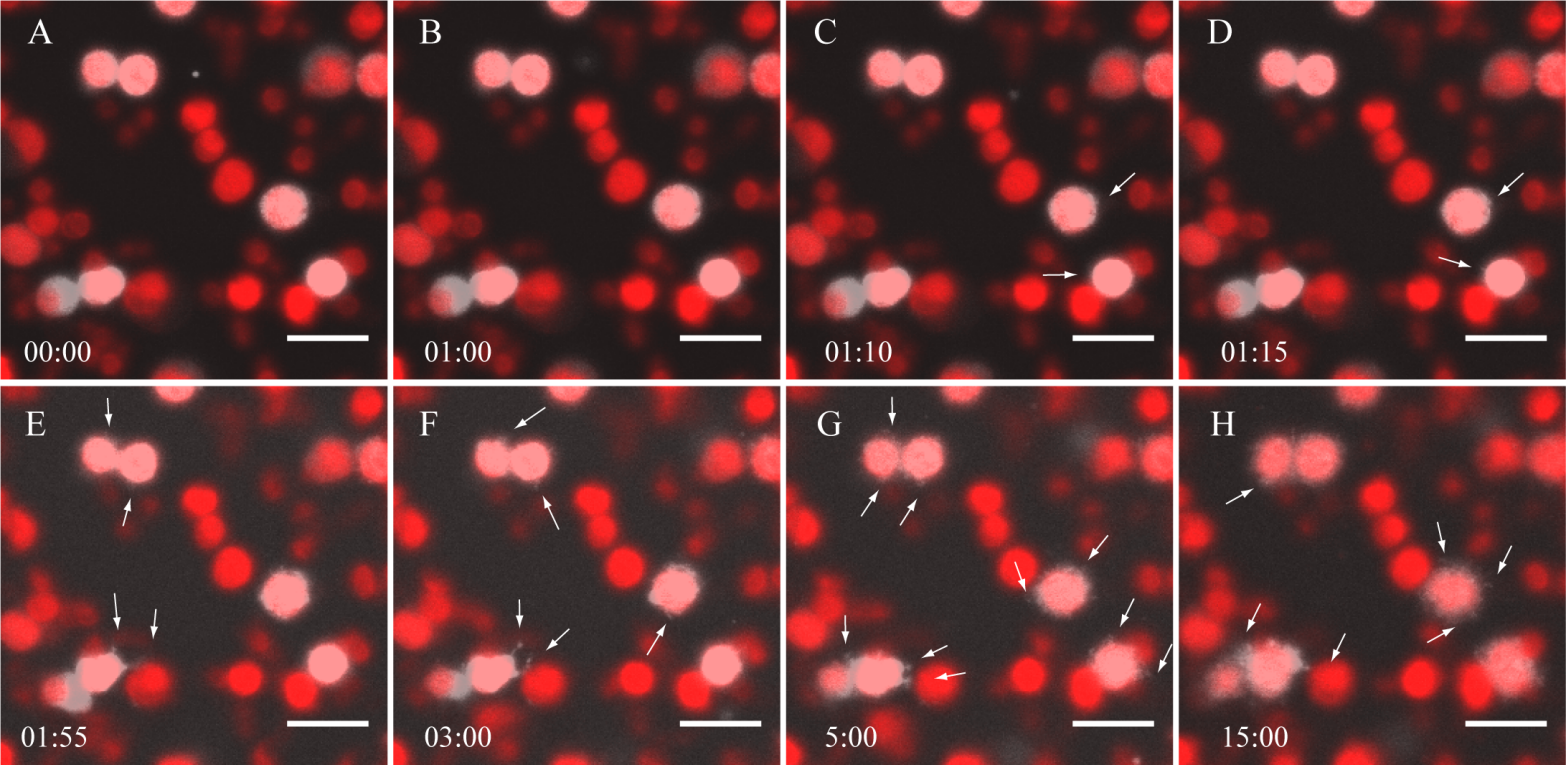
Time-lapse image series of stromule formation *in vitro*. Time (min) is shown in the lower left of each panel. A+B: Image of chloroplasts before the addition of the cell extract. C+D: After 1 minute, the addition of cell extract stimulates stromule formation within ~10 seconds. E+H: Stromules remain present for the remainder of the time course. White arrows mark stromules. The scale bar corresponds to 15 μm.

To measure the kinetics of stromule formation *in vitro* we monitored the changes of chloroplast morphology after cell extract addition in real time. Snapshots lasting 1.7 s were taken at 5 s intervals to prevent photobleaching. The chloroplasts imaged 60 seconds prior to the addition of the cell extract showed a smooth and spherical plastid morphology (Fig 3A and 3B). 1.8% of the total number of chloroplasts (n = 767) observed for these studies displayed short and transient protuberances as seen in the isolated chloroplasts treated with BSA, GTP, and ATP. The earliest observable transformations took place ~10 seconds after the addition of the plant extract, with a few plastids forming small beak-like structures (S4 Movie). By ~ 30 seconds tubules became evident (Fig 3C and 3D, S4 Movie). The stromules were in constant motion throughout the entire imaging process (Fig E-H). Not all the chloroplasts from which stromules eventually emerged displayed these morphological changes immediately, with some starting as long as 240 seconds after the addition of the plant extract. The observed rapid stimulation of stromules further prompted us to perform a preliminary characterization of the stromule-forming compound(s) in the concentrated cell extract.

### Stromule formation is dependent on a protein or protein complex with a molecular mass greater than 100 kDa

**Fig 4:**
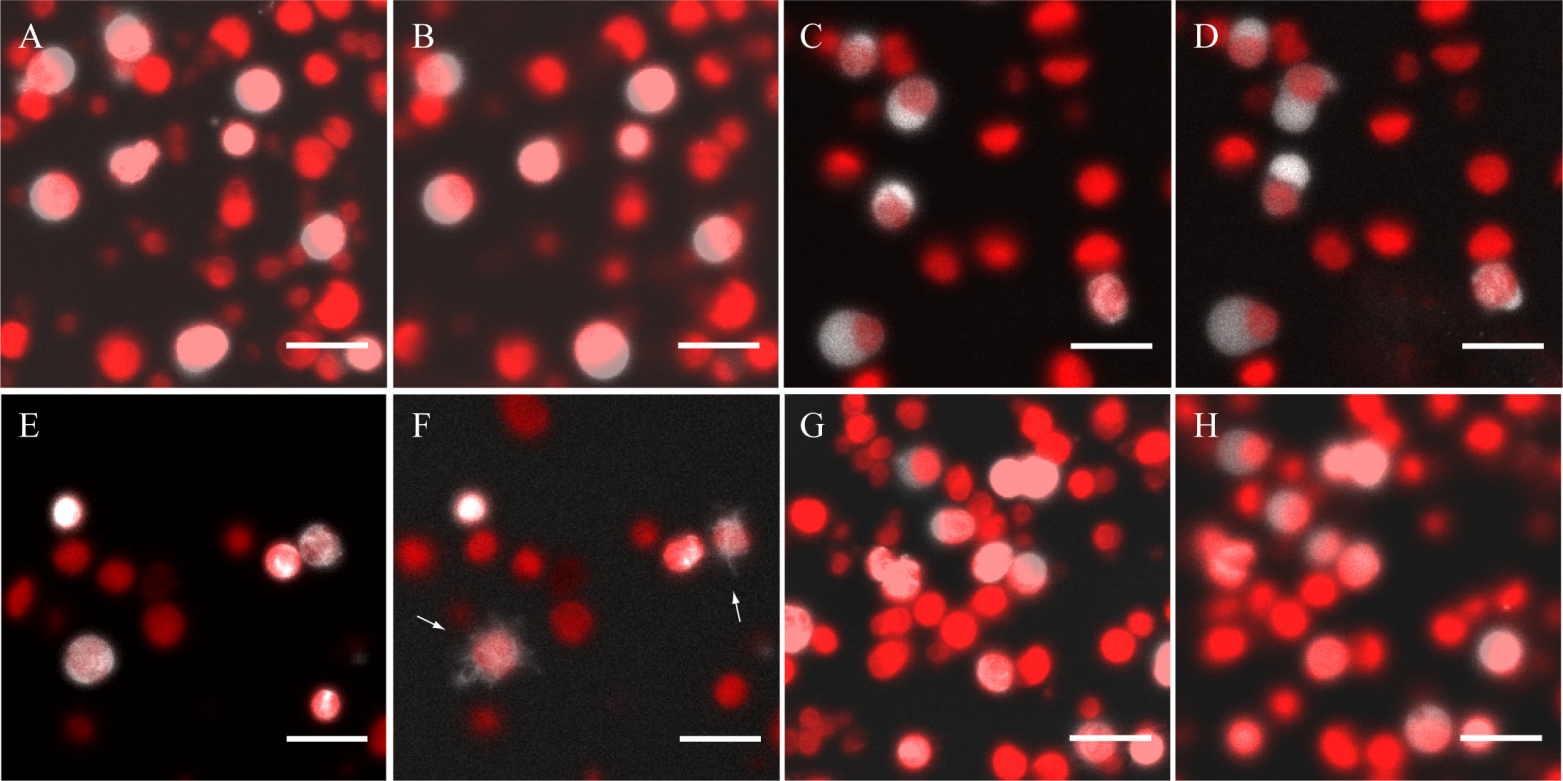
Stromules are stimulated by a 100 kDa or greater sized protein or protein complex. A-B: Images showing isolated chloroplasts before and after the addition of the heat-inactivated cell extract respectively. C-D: Pre and post images of isolated chloroplasts treated with the pellet fraction after a 100,000 x *g* ultracentrifugation of the whole-cell extract, respectively. E-F: Isolated chloroplasts before and after the addition of the supernatant fraction after a 100,000 x *g* ultracentrifugation of the whole-cell extract. G-H: Images of isolated chloroplasts before the addition and after a 15 minute incubation with a cell extract fraction containing proteins smaller than 100 kDa but greater than 10 kDa respectively. Stromules are labeled with white arrows. The scale bar corresponds to 15 μm.

In order to determine if the stromule-stimulating component in the concentrated cell extract is a protein, we attempted to inactivate it by heat. To this end, the plant extract fraction was incubated at 70°C for 10 minutes and the protein aggregates were removed by a brief centrifugation. The remaining plant extract fraction was then transferred into a clean tube and incubated on ice for 30 minutes prior to its addition to the chloroplasts. Compared to the untreated extract, the addition of the heat-inactivated cell extract stimulated the formation of relatively few stromules (10% ± 10.4%, n = 3) (Fig 4A and 4B and S5 Movie). The chloroplasts largely maintained their spherical morphology throughout a 20 minute incubation. The paucity of stromule-forming chloroplasts treated with heat-denatured cell extract suggests that the molecules that stimulates stromule formation is protein based. Ultracentrifugational analysis of the whole-cell extract was utilized to investigate the nature of the stromule-stimulating component. The concentrated extract was centrifuged once at 16,000 x *g* to remove organelles. The supernatant was collected and recentrifuged at 100,000 x *g* for 1 hour to remove proteolipid membrane fragments. Addition of the resuspended proteolipid membrane fraction to isolated chloroplasts did not result in stromule formation (0% ± 0%, n = 5) (Fig 4C and 4D). The addition of the supernatant of the ultracentrifuged whole-cell extract to isolated chloroplasts stimulated stromule formation to levels similar to the non-fractionated cell-extract (38% ± 3.2%, n = 6) (Fig 4E and 4F). The identical stimulatory effect of the supernatant from the ultracentrifuged whole-cell extract suggests that the stromule-forming component is a soluble protein. Since the stromule-stimulating plant extract was concentrated above the 100 kDa cutoff filter, the protein(s) or complex responsible for the stimulation must be at least this large. To confirm this we also tested a fraction that passed through the 100 kDa filter and was concentrated above a 10 kDa filter; this only stimulated 5.7% ± 7.7% (n = 2) of the chloroplasts to form stromules (Fig 4G and 4H). This narrows the effort to identify the cytosolic component(s) that induce stromule formation to a protein or protein complex larger than 100 kDa.

### Stromule formation does not require exogenous ATP in the cell extract

**Fig 5:**
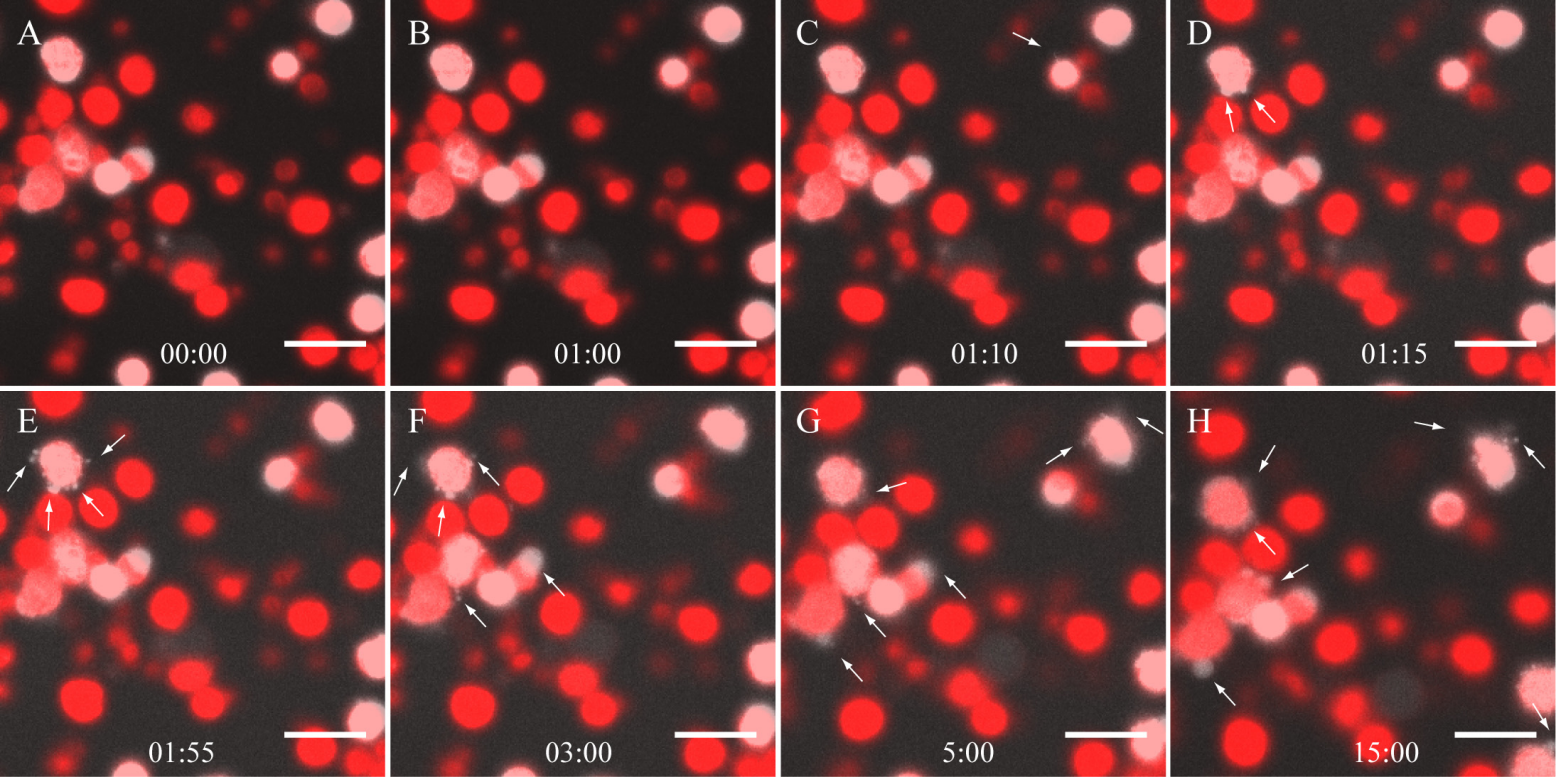
Stromule formation does not require exogenous ATP. A-H: Time-lapse images showing isolated chloroplasts incubated with apyrase-treated cell extract, with duration of the reaction shown in the bottom center of each panel. The apyrase-treated cell extract was added after 1 minute. Stromules are labeled with white arrows. The scale bar corresponds to 15 μm.

Stromule movement has been shown to be dependent on actin filaments and Myosin XI [7, 8, 22]. Myosin XI contains an ATPase domain at the N-terminus that drives the movement of stromules along actin filaments [23]. To determine whether formation as well as movement required ATP we tested the dependence of stromule formation on the presence of ATP by treating the plant extract with 125 units/ml of apyrase for 2 hours on ice. In conjunction with this point, the plant extract was also concentrated 40 fold with a 10 kDa mwco filter, further reducing the amount of ATP present within the plant extract. The addition of the apyrase-treated plant extract fraction to the isolated chloroplasts still stimulated the formation of stromules (51.5% ± 46.0%, n = 2), and with essentially unchanged kinetics (Fig 5A–5H and S6 Movie). Again, the earliest morphological changes took place ~10 seconds after the addition of the extract (Fig 5C and 5D), when a few chloroplasts began to form beak-like projections that eventually turned into short tubules. The number of stromules increased over the course of the 15 minute incubation (Fig 5H). Surprisingly, neither the formation nor the movement of the stromules was inhibited by the apyrase treatment. From these results we conclude that exogenously added ATP is not essential for *in vitro* stromule formation.

## Discussion

Since their rediscovery through the use of GFP and fluorescence microscopy in 1997 [2], stromules have been the subject of more than 60 publications. With but one exception (see below), all of these studies examined stromules *in vivo* or from epidermal peels. The obvious advantages to application of *in vitro* techniques to the elucidation of aspects of stromule formation prompted us to attempt to induce stromule development using isolated chloroplasts. To this end we monitored via confocal microscopy the response to various additions of isolated chloroplasts from *N*. *benthamiana* in which Cerulean is expressed in the stroma. Here we report the first, to our knowledge, observations of stromule formation in intact isolated chloroplasts that is stimulated beyond a low basal level.

In this study, we promoted stromule formation from isolated chloroplasts by the addition of a concentrated plant extract fraction generated during the chloroplast preparation (Fig 1A–1C). Very few isolated chloroplasts displayed transient protuberances (< 0.8 μm) prior to the application of any treatment. Of the total number of isolated chloroplasts observed in the study, only 1.1% of the chloroplasts spontaneously exhibited transient stromule formation, and this served as a baseline of stromule-forming activity from isolated chloroplasts. The stromules formed at the basal level tended to be short and transient in nature. Though the underlying cause of the basal level stromule formation was not further pursued, we note that ER fragments have been shown to remain adherent to the plastid membranes after isolation of intact chloroplasts through a Percoll gradient [24]. It is possible that strong membrane interactions between other proteins or organelles continue after the isolation, and that may contribute towards changes in the organelle morphology.

Stromules were induced by incubating isolated chloroplasts with an undefined cell extract fraction containing elements larger than 100 kDa (Fig 1A–1C). The *in vitro-*constituted stromules reached lengths up to 110 μm, with bulbous structures that appeared asymmetrically within the tubule (Fig 1G–1I). Similar structural characteristics of stromules have also been observed in epidermal peel studies and *in vivo* [6, 10]. We note that, as observed in numerous *in vivo* studies [3, 5, 10], not all isolated chloroplasts gave rise to stromules, while other plastids produced multiple stromules. Approximately 40% of the intact chloroplasts were stimulated by the plant extract to form stromules, a 40 fold increase in stromule formation compared to chloroplasts before plant extract treatment. These observations suggest that our *in vitro* constitution assay successfully recapitulated a number of aspects of stromule formation in live cells. Treatment of plastids with BSA and NTP's (Fig 1D–1F) only minimally increased the occurrence of stromule formation, from ~1% to ~2%. This result suggests that a specific substance within the plant extract is responsible for the formation of stromules. Interestingly, depletion of the plant extract of ATP did not inhibit its ability to induce stromule development.

The change in chloroplast morphology, including the generation of stromules, resulting from the addition of plant extract raises many questions about stromule induction. It has been suggested that stromules arise as a result of failed chloroplast photorelocation away from or towards a light stimulus [25, 26]. We do not favor this hypothesis, however, because while we did notice negative effects of illumination on stromule formation, we observed stromules emanating in all accessible directions (± *x*, ± *y*, + *z*) from plastids fixed in place onto coverslips through poly-lysine interactions (Fig 3, Movie 2). The movement of chloroplasts and stromules have been shown to require actin filaments and myosin [6–8, 22, 27, 28]. Yet, chloroplasts incubated with apyrase-treated cell extract formed stromules at a similar rate as those incubated with ATP present in the cell extract (Fig 4E–4L). It is noteworthy that although the cell extract was depleted of ATP by apyrase treatment, the plastids were not. Accordingly, if ATP is required for *in vitro* stromule formation, it must be utilized either in the chloroplast stroma, or less likely, in the inter-membrane space before it had a chance to equilibrate across the porous outer envelope membrane. This might be consistent with a mechanism suggested by Hanson and Sattarzadeh [29] in which stromules are proposed to be formed by internal pressure, albeit with an additional contribution by factors in the cytoplasm. Recent work by Caplan et al. [14] showed that the knock down of CHUP1, an outer envelope protein known to associate with actin filaments, expression caused constitutive stromule expression. This result suggests that CHUP1 is not an essential protein for stromule formation and that the association of actin with the chloroplast membrane is not crucial for generating the membrane protrusions *in vitro* [14].

Destruction of the plant extract component required for stromule formation by heat, and retention during concentration above a 100 mwco filter suggests that this component is a protein or protein complex with a molecular weight of 100 kDa or greater (Fig 4A–4H). This idea was further supported by the observation that the removal of membrane fragments from the whole-cell extract by ultracentrifugation did not alter the stimulatory behavior of the extract (Fig 4C–4F). These results negate the possibility that the observed chloroplast protrusions are caused by incorporating other protein-lipid fragments into the chloroplast envelope membranes. Stromules have been reported to interact with a number of different organelles, including the ER, nucleus, and mitochondria [6, 8, 17, 30]. A recent mutational analysis of RWP8.2, a protein involved in the detection of specific pathogen effectors, showed that several mutational variants localized to an area that surrounded the stromule membranes [31]. The authors suggested that this layer surrounding the stromules resulted from the rapid exchange of proteins and lipids between the ER and chloroplast, creating conduits for proteins to reach other areas in the cell. The close contact between stromules and different organelles suggests that the immediate environment surrounding the stromules may play a significant role in contributing proteins to their formation.

The generation of tubules from artificial membrane bilayers *in vitro* may provide some clues to understanding the formation of stromules [32–34]. These studies have raised the possibility that stromule formation is mediated by a dynamin-related protein. Plant dynamins can be categorized into 4 subfamilies, with proteins ranging from 68 kDa to 100 kDa [35, 36]. The addition of human neural dynamin to DOPC:DOPS:PI4,5-P_2_ lipid bilayers resulted in tubulation of the artificial bilayer after the addition of GTP [32]. The projections had diameters of ~0.5 μm and reached lengths of up to 30 μm, not unlike that observed for stromules. The rate of extension also occured on a similar time scale as that we observed for stromules in our study.

Another possible contribution to stromule formation could be the over-crowding of proteins in or along the envelope membranes. A recent study showed that proteins involved in clatharin-mediated endocytosis, epsin1 and AP180 induced vesicle tubulation through the steric pressure generated by having a high density of proteins linked to the membrane [33]. The tubulation was dependent on the carrier protein size and its concentration along the membrane. Protein crowding-induced membrane tubulation has also been reported in chloroplasts in which outer envelope membrane proteins had been overexpressed [37–39]. The stromule-like protrusions were thought to be caused by an increased concentration of membrane proteins and appeared to be independent of protein size and function. Machettira et al. [37] showed that GFP fused to the transmembrane domain of CHUP1 induced membrane protrusions, which further supports the finding that CHUP1 itself is not crucial for stromule formation. In light of this result, the proliferation of stromules following the reduction of CHUP1 expression demonstrated by Caplan et al. [14] could be explained if removal of CHUP1 made room for the accumulation of other outer envelope proteins that function to alter membrane curvature. Additionally, Caplan et al. showed that overexpression of a CHUP1 transit peptide-RFP chimera impaired the chloroplasts’ ability to form stromules. This overexpressed chimera also saturated the chloroplast import machinery, although it remains unclear whether stromule formation *in vivo* is linked to plastid protein import; our *in* vitro experiments suggest it is not. In aggregate, these studies suggest that outer envelope membrane proteins play a pivotal role in the formation of these protrusions.

While this manuscript was under revision a new study reporting stromule formation by isolated chloroplasts appeared in the literature [40]. The stromules formed *in vitro* in the Brunkard et al. study were often short and occurred at a low frequency, and most closely resembled structures that we classified in our study as small stromules forming at a basal level in isolated chloroplasts. We speculate they may be formed under the influence of additional proteins and membranes that remain in contact with the chloroplasts after isolation. In contrast to Brunkard et al. [40], we observed an approximately 40-fold stimulation in stromule occurrence, which appeared rapidly and often became quite long, upon addition of a concentrated cell extract containing elements, probably proteins, retained by a 100 kDa mwco filter. The reasons for the discrepancies between these two studies is currently unknown.

The development of the *in vitro* stromule formation assay reported here will allow for the testing of models governing stromule development, and opens a path toward identification of the protein(s) required for this process. This effort is currently ongoing in our lab. We anticipate that this new stromule assay will lead to a better understanding of the mechanism of formation and purpose of these enigmatic dynamic plastidic structures.

## Supporting Information

S1 Movie Stromule extension over long distances. A video showing a stromule emanating from a chloroplast body that is 109 nm in length. Scale bars correspond to 30 μm. The white arrow corresponds to a stromule.
(AVI)

S2 Movie Stromule branching. A video panning out of a stromule that is branched. The white arrow indicates an area of stromule branching.
(AVI)

S3 Movie Video of *in vitro* formed stromules in solution. A video capturing the recoiling of stromules back to the main plastidic body. Scale bars correspond to 10 μm.
(AVI)

S4 Movie Video of *in vitro* stromule formation. A video showing the formation of stromules in real-time. The white arrows indicate chloroplasts that are stimulated by the cell extract to form stromules. Scale bars correspond to 30 μm. The total elapsed time of the recording was 20 minutes.
(AVI)

S5 Movie Heat-inactivated cell extract addition to isolated chloroplasts. A video showing the captured stimulation of stromules from the heat inactivated plant extract. Scale bars correspond to 30 μm. The total elapsed time of the recording was 20 minutes.
(AVI)

S6 Movie Video of stromule formation after the addition of apyrase-treated cell extract. A video showing the formation of stromules after the addition of apyrase-treated cell extract. The white arrows correspond to chloroplasts that form stromules. Scale bars correspond to 30 μm. The total elapsed time of the recording was 20 minutes.
(AVI)
